# Temporal regulation of mRNAs for select bone morphogenetic proteins (BMP), BMP receptors and their associated SMAD proteins during bovine early embryonic development: effects of exogenous BMP2 on embryo developmental progression

**DOI:** 10.1186/1477-7827-12-67

**Published:** 2014-07-15

**Authors:** Kyung-Bon Lee, Joseph K Folger, Sandeep K Rajput, George W Smith

**Affiliations:** 1 Department of Laboratory of Mammalian Reproductive Biology and Genomics, Michigan State University, East Lansing, MI 48824, USA; 2 Department of Animal Science, Michigan State University, East Lansing, MI 48824, USA; 3 Department of Physiology, Michigan State University, East Lansing, MI 48824, USA; 4 Department of Biology Education, College of Education, Chonnam National University, Gwangju 500-757, Republic of Korea

**Keywords:** Ovary, Bovine, Oocyte, Embryo, Blastocyst, BMP, BMP receptor, SMAD

## Abstract

**Background:**

We previously demonstrated embryotrophic actions of maternal (oocyte-derived) follistatin during bovine early embryogenesis. Classical actions of follistatin are attributed to inhibition of activity of growth factors including activins and bone morphogenetic proteins (BMP). However, temporal changes in BMP mRNA in early bovine embryos and the effects of exogenous BMP on embryo developmental progression are not understood. The objectives of present studies were to characterize mRNA abundance for select BMP, BMP receptors and BMP receptor associated SMADs during bovine oocyte maturation and early embryogenesis and determine effects of addition of exogenous BMP protein on early development.

**Methods:**

Relative abundance of mRNA for *BMP2*, *BMP3*, *BMP7*, *BMP10*, *SMAD*1, *SMAD5*, *ALK3*, *ALK6*, *ALK2*, *BMPR2*, *ACVR2A* and *ACVR2B* was determined by RT-qPCR analysis of germinal vesicle (GV) and in vitro matured metaphase II (MII) oocytes and in vitro produced embryos collected at pronuclear, 2-cell (C), 4C, 8C, 16C, morula and blastocyst stages. Effects of addition of recombinant human BMP2 (0, 1, 10 and 100 ng/ml) during initial 72 h of embryo culture on early cleavage (within 30 h post insemination), total cleavage, development to 8C-16C and blastocyst stages and blastocyst mRNA abundance for markers of inner cell mass (*NANOG*) and trophectoderm (*CDX2*) were also determined.

**Results:**

Abundance of mRNA for *BMP2*, *BMP10*, *SMAD1*, *SMAD5*, *ALK3*, *ALK2*, *BMPR2* and *ACVR2B* was elevated in MII oocytes and/or pronuclear stage embryos (relative to GV) and remained elevated through the 8C -16C stages, whereas *BMP3*, *BMP7* and *ALK2* mRNAs were transiently elevated. Culture of embryos to the 8C stage in the presence of α-amanitin resulted in increased abundance for all of above transcripts examined relative to untreated 8C embryos. Effects of addition of exogenous BMP2 on early cleavage rates and rates of development to 8C-16C and blastocyst stages were not observed, but BMP2 treatment increased blastocyst mRNA for *CDX2* and *NANOG*.

**Conclusions:**

Abundance of maternally derived mRNAs for above BMP system components are dynamically regulated during oocyte maturation and early embryogenesis. Exogenous BMP2 treatment does not influence progression to various developmental endpoints, but impacts characteristics of resulting blastocysts. Results support a potential role for BMPs in bovine early embryogenesis.

## Background

Oocyte developmental competence was defined by Sirard *et al.*[1] as the capacity of the oocyte to resume meiosis, cleave after fertilization, help promote embryonic development and implantation, and bring a pregnancy to term in good health [1]. Our previous studies support a positive functional role for maternal (oocyte-derived) follistatin in bovine oocyte competence. Follistatin mRNA is positively associated with developmental competence in two distinct bovine models of egg quality [2,3]. Furthermore, follistatin supplementation during the first 72 h of bovine embryo culture (until embryonic genome activation) enhanced proportion of embryos that cleaved early and proportion of embryos developing to the blastocyst stage in a dose dependent fashion [3]. Follistatin treatment also increased total blastocyst cell numbers specifically through an increase in trophectoderm (TE) cells, with no effect on numbers of inner cell mass (ICM) cells and increased blastocyst mRNA for the TE specific transcription factor *CDX2*[3]. We also observed similar effects of follistatin treatment on early cleavage and rates of development to blastocyst stage for rhesus monkey embryos [4], demonstrating potential translational relevance of results from the bovine model system. However, the mechanisms responsible for stimulatory effects of follistatin on multiple indices of bovine early embryonic development to date still remain elusive.

Follistatin was initially classified as a high affinity binding protein inhibiting activin action [5]. However, stimulatory effects of exogenous activin treatment on early cleavage and blastocyst rates for bovine embryos were also observed suggesting a potential alternative mechanism of follistatin action [3]. Follistatin can also bind and regulate activity of multiple additional TGFβ superfamily members including BMPs [6-8]. A prominent role for BMPs in regulation of patterning of early embryos has been described [9]. Furthermore, BMPs have been implicated in regulation of trophoblast differentiation in human embryonic stem cells [10]. However, less is known about their role during preimplantation embryonic development, particularly in farm species.

In this study, we investigated temporal changes in mRNA abundance for multiple BMP, BMP receptors and their associated SMADs in bovine oocytes and embryos and the source of such transcripts (maternal versus embryonic) in bovine embryos coincident with embryonic genome activation. We also determined the effect of exogenous BMP2 on multiple endpoints relevant to bovine early embryonic developmental progression. Results demonstrate dynamic regulation of maternal mRNAs for BMP system components during bovine early embryonic development and effects of exogenous BMP2 treatment on indices of cell lineage determination in bovine blastocysts. Results also suggest embryotropic actions of follistatin on bovine early embryogenesis are likely not mediated by antagonism of BMP2 signaling.

## Methods

### Oocyte collection, in vitro maturation, in vitro fertilization and embryo culture

Bovine oocytes used for all described experiments were obtained from ovaries harvested at a local abattoir. Aspiration and in vitro maturation of oocytes, in vitro fertilization and culture of embryos was performed as previously described [3]. Cumulus-oocyte complexes (aspirated from 2–7 mm visible follicles) with > 4 compact cumulus cell layers and homogeneous cytoplasm were matured in TCM 199 [supplemented with 0.2 mM sodium pyruvate, 5 mg/ml gentamicin sulfate, 6.5 mM L-glutamine, 156 nM bovine LH (Sioux Biochemical, Sioux Center, Iowa), 15.6 nM bovine FSH (Sioux Biochemical), 3.67 nM 17β-estradiol and 10% v/v defined FBS (Hyclone, Logan, UT)] for 24 h at 38.5°C, 5% CO_2_ in air with maximum humidity. For in vitro fertilization, matured oocytes were washed and co-incubated with sperm for 20 h in fertilization medium (114 mM NaCl, 25 mM NaHCO_3_, 3.2 mM KCl, 0.34 mM NaH_2_PO_4_, 0.183 mM penicillin-G, 16.6 mM sodium lactate, 0.5 mM MgCl_2_·6H_2_O, 2.7 mM CaCl_2_·2H_2_O, 0.2 mM sodium pyruvate, 6 mg/ml BSA and 1.5 U of heparin) at 38.5°C, 5% CO_2_ in air with maximum humidity. After cumulus cell removal, presumptive zygotes were washed and cultured in potassium simplex optimization medium (KSOM; EMD Millipore, Billerica MA) supplemented with 0.3% BSA for 72 h. Embryos were then washed and cultured in fresh KSOM medium supplemented with 0.3% BSA and 10% FBS until day 7.

### Temporal changes in BMP system mRNA abundance in bovine oocytes and early embryos

Germinal vesicle (GV) and metaphase (MII) stage (oocytes), pronuclear (PN), 2-cell (C), 4C, 8C, 16C, morula and blastocyst stage embryos (n = 4 pools of 10 each), were collected as we described previously [3,11]. For GV and MII stage oocyte RNA samples, cumulus cells were completely removed by hyaluronidase (0.1%) digestion and repeated pipetting and denuded oocytes in groups of 10 each were snap frozen in 100 μl lysis solution (RNAqueous Micro Kit, Ambion Inc, Austin, TX) and stored at -80°C until RNA isolation. Embryo samples were also processed as described above in groups of 10 per sample per stage with PN embryos harvested at 20 h post insemination (hpi), 2C embryos collected 33 hpi, 4C embryos 44 hpi, 8C embryos 52 hpi, 16C embryos 72 hpi, and morulas and blastocysts at 5 and 7 d post insemination respectively. 250 femtograms of polyadenylated GFP RNA were added to each sample prior to RNA extraction. After finishing RNA isolation and cDNA synthesis, cDNA was diluted 1:5 and mRNA quantification for *BMP2*, *BMP3*, *BMP7*, *BMP10*, *ALK2*, *ALK3*, *ALK6*, *BMPR2*, *ACVR2A*, *ACVR2B*, *SMAD1* and *SMAD5* mRNAs was performed in duplicate for each sample by qPCR using our procedures reported previously [11]. Relative expression levels were calculated using the ΔΔCT method with *RPS18* as the housekeeping gene [12], *RPS18* mRNA abundance is very stable across MII through 16C stages and then is increased in later stages coincident with increase in cell numbers (Additional file 1: Figure S1). Abundance of exogenous control (GFP) RNA was also measured to account for variation in RNA recovery and cDNA synthesis across samples and exogenous GFP mRNA abundance was similar (P > 0.2) across samples. Primer sequences and fragment sizes for all transcripts measured are included in Table 1 and PCR efficiencies for all primer sets were between 90 and 103%.

**Table 1:** **Sequence of primers for real time RT-PCR for TGF**β **superfamily members and receptors, *CDX2 *** **and *NANOG***

**Gene**	**Genbank accession number**	**Primer sequence**	**Size (bp)**
*BMP2*	BC134682	F: 5′-AAGGCCCTTGCTTGTCACTTT-3′	72
		R: 5′-TGCTTGCCGCTTTTCTCTTC-3′	
*BMP3*	XM_587912	F: 5′-ATCTGTGGCTGAGCTGCTTGT-3′	62
		R: 5′-GGAAGGGCTGCCTGAGTCT-3′	
*BMP7*	XM_612246	F: 5′-TGCCACTAGCTCTTCCTGGAA-3′	65
		R: 5′-TGAGAGACCCAGGATCCAGAA-3′	
*BMP10*	XM_583418	F: 5′-CGCCCACGAGCAATTCC-3′	66
		R: 5′-TCCCCAGGTCCGTTGGA-3′	
*SMAD1*	BC116117	F: 5′-CACCATGAACTGAAACCATTGG-3′	68
		R: 5′-GATGCACACCTCCTTCTGCTT-3′	
*SMAD5*	DV821574	F: 5′-GCAACGTTTCCTGATTCTTTCC-3′	74
		R: 5′-GGCGGGTAGGGACTATTTGG-3′	
*ALK3*	NM_001076800	F: 5′-TCAGCGAACTATTGCCAAACAG-3′	75
		R: 5′-CCCATCCACACTTCTCCGTATC-3′	
*ALK6*	NM_001105328	F: 5′-CCCACCCCTCGTCCAAAG-3′	63
		R: 5′-GACCGAGTCTTCTGGACAATGG-3′	
*ALK2*	BC133311	F: 5′-TTGGCCTCATCATTTTGTCTGT-3′	70
		R: 5′-CGGAGAGCAACTCCCAATAGG-3′	
*ACVR2A*	NM_174227	F: 5′- CCACAAACCCGCCATATCTC -3′	185
		R: 5′- TAGCACCCTCTAACACCTCTG -3′	
*ACVR2B*	NM_174495	F: 5′-GGAGCCATCAACTTCCAGAG-3′	121
		R: 5′-GCATGTACTCATCCACAGGTC-3′	
*BMPR2*	XM_002685492	F: 5′-AACACCACTCAGTCCACCTC-3′	120
		R: 5′-GTCAGCATCCTATATCCAAAGCA-3′	
*NANOG*	NM_001025344	F: 5′-AAAGTTACGTGTCCTTGCAAACG-3′	73
		R: 5′-GAGGAGGGAAGAGGAGAGACAGT-3′	
*CDX2*	AM293662	F: 5′-FCGTCTGGAGCTGGAGAAGGA-3′	70
		R: 5′-CGGCCAGTTCGGCTTTC-3′	

### Effect of transcriptional inhibition on BMP system mRNA abundance

To determine the effects of inhibition of transcription on mRNA abundance in in vitro derived embryos, presumptive zygotes were cultured in serum free KSOM with 0.3% BSA with or without the addition of 50 μg/ml α-amanitin (Sigma, St. Louis, MO). The 8C embryos were then collected at 52 h post insemination (n = 4 pools of 10 embryos per group) and placed in lysis buffer and snap frozen and stored as above until RNA isolation. Half the RNA was subjected to reverse transcription using oligo(dT) primers as described above for use in quantification of polyadenylated transcripts for genes of interest. The remaining RNA was transcribed using random hexamers for quantification of adenylated and deadenylated (total) transcripts for genes of interest. The cDNA produced was subjected to qPCR for the BMP system components as described above. Data were normalized relative to abundance of endogenous control (*RPS18*).

### Effect of BMP2 supplementation on bovine embryo developmental progression

To examine the effect of BMP2 supplementation on early cleavage rate (assessed 30 hpi), rate of development to 8C-16C stage (assessed 72 hpi), blastocyst rate (assessed 7 d post insemination) and the abundance of mRNA for the TE marker (*CDX2*) and ICM marker (*NANOG)* in resulting blastocysts, presumptive zygotes were cultured in KSOM medium supplemented with 0.3% BSA containing 0, 1, 10 or 100 ng/ml BMP 2 (30 presumptive zygotes per group, 4 replicates). The 8C-16C stage embryos were then separated 72 h post fertilization and cultured in fresh KSOM medium (minus exogenous BMP2) supplemented with 0.3% BSA and 10% FBS until d 7. Blastocysts were harvested at d 7 post fertilization (n = 4 pools of 5 blastocysts each per treatment) and lysed and frozen as above until RNA isolation and RT-qPCR analysis as described above.

### Statistical analysis

All data were analyzed using one way ANOVA in SAS followed by Fishers Protected Least Significant Difference Test to determine differences between means. For embryo culture experiments, % data were arc-sin transformed prior to analysis. Data are presented as mean ± SEM.

## Results and discussion

### Temporal regulation of BMP mRNA abundance during oocyte maturation and early embryogenesis

**Figure 1:**
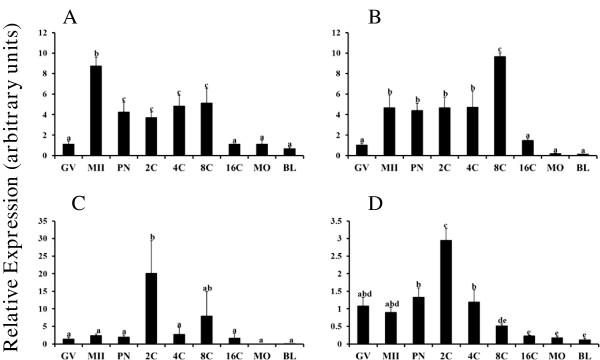
**Temporal changes in *BMP2 *(A), *BMP10 *(B), *BMP3 *(C) and *BMP7 *(D) mRNA abundance during oocyte maturation and early embryogenesis in vitro.** Quantitative real time RT-PCR analysis was performed on samples of germinal vesicle (GV) and metaphase II (MII) stage oocytes and in vitro derived embryos collected at the pronuclear (PN), 2-cell (2C), 4-cell (4C), 8-cell (8C), 16-cell (16C), morula (MO) and blastocyst (BL) stages (n = 4 pools of 10 oocytes/embryos per pool). Data were normalized relative to abundance of endogenous control (*RPS18*) and are shown as mean ± SEM. Values with different superscripts across timepoints denote significant differences (P < 0.05).

Transcriptome analysis of human oocytes indicates that multiple key components of the TGFβ superfamily signaling pathway are potentially active [13] and previous studies support a functional role for TGFβ superfamily members during bovine oocyte maturation and early embryogenesis [3,14,15]. Over 20 members of the BMP subfamily have been described [16], and expression of BMP in the bovine ovary has been extensively studied [17-19]. Abundance of specific mRNA transcripts during oocyte maturation and early embryonic development is under complex regulation and influenced by post transcriptional and transcriptional mechanisms in a stage specific fashion [20]. Results of present studies revealed unique temporal changes in mRNA abundance for above BMP examined during oocyte maturation and early embryogenesis (Figure 1). Relative abundance of mRNA for *BMP2* (Figure 1A) and *BMP10* (Figure 1B) was increased in MII oocytes relative to the GV stage (P < 0.05), but *BMP3* (Figure 1C) and *BMP7* (Figure 1D) mRNA abundance did not change during meiotic maturation. For *BMP2* and *BMP10*, mRNA abundance remained elevated in early embryos after fertilization until declining by the 16C stage (P < 0.05) and remained low in morula and blastocyst stage embryos (Figure 1A and B). In contrast, relative mRNA abundance for *BMP3* was transiently elevated (>15 fold) in 2C embryos (P < 0.05) and did not differ at other time points examined (Figure 1C). *BMP7* mRNA (Figure 1D) was also transiently elevated at the 2C stage and was lowest at 16C, morula and blastocyst stages (P < 0.05). Results demonstrate dynamic, ligand specific temporal regulation of mRNA abundance for *BMP2*, *BMP3*, *BMP7* and *BMP10* during bovine oocyte maturation and early embryogenesis.

**Figure 2:**
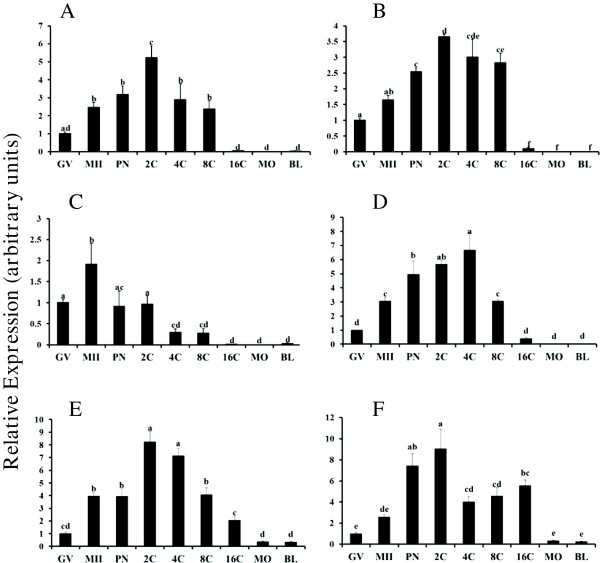
**Temporal changes in *ALK3 *(A), *ALK6 *(B), *ALK2 *(C) *BMPR2 *(D), *ACVR2B *(E) and *ACVR2A *(F) mRNA abundance during oocyte maturation and early embryogenesis in vitro.** Quantitative real time RT-PCR analysis was performed on samples of germinal vesicle (GV) and metaphase II (MII) stage oocytes and in vitro derived embryos collected at the pronuclear (PN), 2-cell (2C), 4-cell (4C), 8-cell (8C), 16-cell (16C), morula (MO) and blastocyst (BL) stages (n = 4 pools of 10 oocytes/embryos per pool). Data were normalized relative to abundance of endogenous control (*RPS18*) and are shown as mean ± SEM. Values with different superscripts across time points denote significant differences (P < 0.05).

The BMPs bind to multiple TGFβ superfamily receptors [16]. Expression of BMP receptors in bovine ovarian sections and or isolated oocytes has been reported previously [19], but temporal regulation of mRNA abundance for BMP receptors during oocyte maturation and early embryogenesis is not understood. In the current study, temporal changes in abundance of mRNA in bovine oocytes and early embryos for the prominent Type I (*ALK3*, *ALK6*, *ALK2*) and Type II receptors (*BMPR2*, *ACVR2A*, *ACVR2B*) that bind BMPs was examined (Figure 2). Abundance of mRNA for *ALK3* (Figure 2) was increased in MII oocytes (relative to GV stage), further increased, albeit transiently at the 2C stage, decreased in 4C and 8C embryos and further decreased in 16C, morula and blastocyst stage embryos (P < 0.05). In contrast, *ALK6* mRNA (Figure 2B) is elevated at the PN stage, further increased, albeit transiently at the 2C stage and decreased at 16C, morula and blastocyst stages (P < 0.05). *ALK2* mRNA (Figure 2C) is transiently elevated in MII oocytes relative to oocytes collected at the GV stage, and reduced even further in embryos collected at 4C stage and beyond (P < 0.05). For the type II receptors, *BMPR2* (Figure 2D) is increased in MII oocytes relative to GV stage, further increased post fertilization through the 4C stage and subsequently declines through 16C stage (P < 0.05). While mRNA for *ACVR2B* (Figure 2E) but not *ACVR2A* (Figure 2F) was increased in MII oocytes relative to GV stage, mRNA for both *ACVR2B* and *ACVR2A* was elevated at PN stage and remained elevated through the 16C stage, and was further reduced at morula and blastocyst stages (P < 0.05; Figure 2E and F). Observed temporal regulation of type I and Type II BMP receptor mRNAs supports a potential intrinsic role for BMPs in meiotic maturation and early embryogenesis.

**Figure 3:**
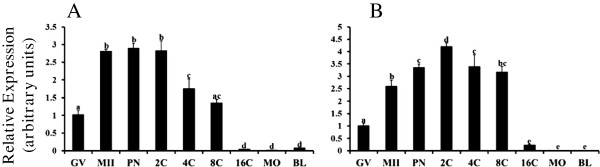
**Temporal changes in *SMAD1 *(A) and *SMAD5 *(B) mRNA abundance during oocyte maturation and early embryogenesis in vitro.** Quantitative real time RT-PCR analysis was performed on samples of germinal vesicle (GV) and metaphase II (MII) stage oocytes and in vitro derived embryos collected at the pronuclear (PN), 2-cell (2C), 4-cell (4C), 8-cell (8C), 16-cell (16C), morula (MO) and blastocyst (BL) stages (n = 4 pools of 10 oocytes/embryos per pool). Data were normalized relative to abundance of endogenous control (*RPS18*) and are shown as mean ± SEM. Values with different superscripts across time points denote significant differences (P < 0.05).

Signal transduction by members of the TGFβ superfamily is mediated primarily by the SMAD pathways, with BMP signal transduction linked to SMAD1, SMAD5 and SMAD8 [16]. Thus changes in mRNA for BMP receptor associated SMADs during oocyte maturation and early embryogenesis were also investigated (Figure 3). Messenger RNA for *SMAD8* was undetectable in bovine oocytes and early embryos. Messenger RNA for *SMAD1* (Figure 3A) and *SMAD5* (Figure 3B) were increased in MII oocytes relative to GV stage (P < 0.05). For *SMAD1*, mRNA remained elevated after fertilization until the 4C stage and was further decreased in 16C, morula and blastocyst stage embryos (P < 0.05; Figure 3A). For *SMAD5*, mRNA was further increased after fertilization at PN, 2C and 4C stages and lowest at 16C, morula and blastocyst stages (P < 0.05; Figure 3B).

### Effect of embryo culture in the presence of the transcriptional inhibitor α-amanitin on BMP, BMP receptor and SMAD1 and SMAD5 mRNA abundance

Messenger RNA transcript adenylation and deadenylation are prominent mechanisms of regulation of mRNA abundance during oocyte maturation and early development until initiation of embryonic genome activation [20,21]. Given abundance of above BMP, BMP type I receptor and SMAD transcripts was elevated in early embryos prior to or through the 8C stage, studies were done to determine the source (maternal versus embryonic) of *BMP2*, *BMP3*, *BMP7*, *BMP10*, *SMAD1*, *SMAD5*, *ALK2*, *ALK3* and *ALK6*, transcripts detected in early bovine embryos. When presumptive zygotes were treated with 50 μg/ml of the RNA polymerase II inhibitor α-amanitin for 72 h, relative abundance of mRNA for all BMP system components examined was increased at the 8C stage compared to control embryos (Table 2). Results suggest that such transcripts present in early embryos at 8C stage are maternal in origin and that post transcriptional regulation of mRNA abundance for such genes may in fact be transcription dependent. Furthermore elevated transcript abundance was observed when reverse transcription of mRNA was conducted using oligo dT primers and not when random hexamers were utilized (Table 2), suggesting that transcript deadenylation may help contribute to the stage specific decrease in transcript abundance for BMP, BMP receptors and their receptor associated SMAD in the current studies. Since removal of the poly-A tail inhibits translation and is a starting point for RNA degradation [22], results suggests that down regulation of above transcripts may be important during stages of embryo development after embryonic genome activation.

**Table 2:** **Effects of** α**-Amanitin treatment on mRNA abundance in bovine 8C embryos**

**Gene**	**RT with oligo dT**		**RT with random hexamers**	
	**Control**	**α-Amanitin treated**	**Control**	**α-Amanitin treated**
*SMAD1*	1.15 ± 0.27^a^	9.08 ± 1.19^b^	1.03 ± 0.16	1.77 ± 0.17
*SMAD5*	1.17 ± 0.35^a^	7.33 ± 1.27^b^	1.02 ± 0.12	1.45 ± 0.17
*BMP2*	1.10 ± 0.27^a^	3.19 ± 0.76^b^	1.25 ± 0.49	1.30 ± 0.21
*BMP3*	1.02 ± 0.11^a^	2.85 ± 0.60^b^	1.15 ± 0.36	3.26 ± 0.88
*BMP7*	1.02 ± 0.12^a^	3.36 ± 0.66^b^	1.17 ± 0.40	2.96 ± 1.31
*BMP10*	1.04 ± 0.19^a^	3.82 ± 0.25^b^	1.07 ± 0.23	1.59 ± 0.31
*ALK3*	1.23 ± 0.37^a^	9.41 ± 0.72^b^	1.06 ± 0.22	1.42 ± 0.31
*ALK6*	1.12 ± 0.25^a^	5.43 ± 0.71^b^	1.05 ± 0.20	1.38 ± 0.16
*ALK2*	1.27 ± 0.50^a^	5.73 ± 1.34^b^	1.02 ± 0.13	0.86 ± 0.09

*Note.* Different letters (a and b) represent significantly different value (P≤0.05).

### Effect of BMP 2 supplementation on embryo development and trophectoderm and inner cell mass marker mRNA abundance in resulting blastocysts

**Figure 4:**
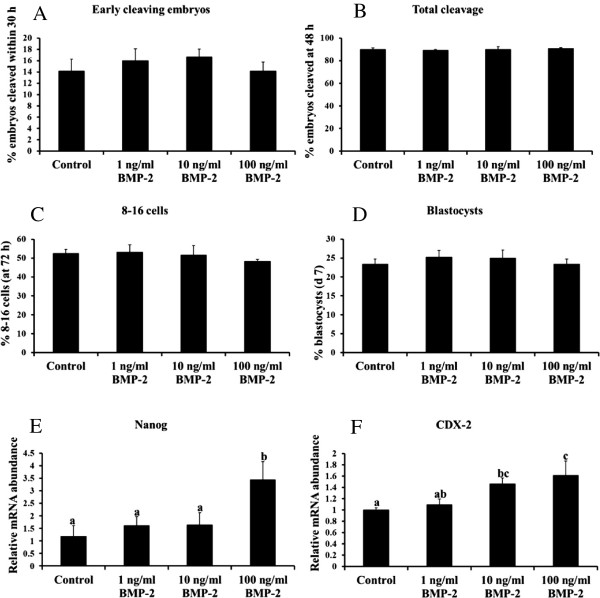
**BMP2 regulation of bovine early embryonic development.** Effects of exogenous BMP2 treatment (0, 1, 10, 100 ng/ml) during first 72 h of *in vitro* embryo culture. (n = 30 embryos/treatment; n = 4 replicates) on proportion of early cleaving embryos that reached the two-cell stage within 30 hours post insemination (hpi) **(A)**, total cleavage rate (determined 48 hpi) **(B)**, rates of development to 8- to 16-cell stage (determined 72 hpi) **(C)** d7 blastocyst rates **(D)** and blastocyst mRNA abundance for *NANOG***(E)** and *CDX2***(F)**. Data are shown as mean ± SEM. Values with different superscripts across treatments indicate significant differences (P < 0.05).

Previous studies demonstrated a positive association of oocyte follistatin expression with developmental competence [2] and potent embryotrophic actions of follistatin during early embryogenesis including enhanced proportion of embryos cleaving early, increased numbers of embryos developing to the blastocyst stage, and elevated mRNA for the TE cell marker *CDX2* in resulting blastocysts [3]. Classical actions of follistatin are commonly attributed to high affinity binding and inhibition of activity of the TGFβ superfamily member activin [5]. However, previous studies showed stimulatory effects of activin treatment on rates of early cleavage and blastocyst development, suggesting that actions of follistatin on bovine embryos are likely nonclassical [3]. However, follistatin can also bind at a lower affinity and inhibit activity of BMPs [7,17]. The current studies demonstrated dynamic temporal changes in maternal mRNA for several BMP, type I and II BMP receptors and their receptor associated SMADs in bovine embryos prior to genome activation. Therefore to further elucidate the potential role of BMPs in bovine early embryogenesis, effects of BMP2 supplementation during first 72 h of in vitro embryo culture (through 8C-16C stage) on above developmental endpoints were investigated. The addition of 1, 10 or 100 ng/ml of recombinant human BMP2 did not alter percentage of early cleaving embryos, total cleavage rates, percent embryos developing to 8C-16C stage or blastocyst rate (Figure 4A-D). However supplementation with 100 ng/ml of BMP2 increased mRNA for the TE marker *CDX2* and the ICM marker *NANOG* relative to embryos cultured without BMP2 (Figure 4E-F). Thus, while BMP2 supplementation did not impact efficiency of bovine in vitro embryonic development as measured by numbers of embryos reaching a transferable (blastocyst stage), BMP2 treatment did however impact characteristics of resulting blastocysts as measured by abundance of mRNA for *CDX2* and *NANOG*. These results are in contrast to results obtained following treatment with exogenous follistatin [3] whereby stimulatory effects on *CDX2* mRNA, but not *NANOG* mRNA were observed and suggesting biological actions of follistatin on blastocyst cell allocation are manifest specifically on the TE lineage.

A growing body of evidence suggests that oocyte secreted factors can enhance oocyte developmental competence and embryo developmental progression [14,15,23]. Our previous studies support a role for endogenous, oocyte-derived follistatin in promoting bovine embryo developmental progression and cell allocation to the TE lineage [3]. Results were obtained with follistatin treatment during initial stages of embryo culture (d 1–3) up to embryonic genome activation. To our knowledge, effects of exogenous BMP2 treatment during culture of bovine embryos have not been previously reported. Addition of BMP2 or BMP4 during in vitro maturation did not impact rates of meiotic maturation and cumulus expansion or rates of embryonic development to blastocyst stage following in vitro fertilization [19]. However, evidence supports a role for oocyte-derived GDF9 and BMP15 in promoting oocyte developmental competence as addition of these growth factors exogenously during in vitro maturation can enhance rates of development to the blastocyst stage and blastocyst cell allocation to TE [14,15]. Thus, levels of endogenous BMP2 available during in vitro maturation and embryo culture may not be limiting to embryo developmental capacity in vitro as reflected by rates of development to the blastocyst stage. However, the current studies do demonstrate that bovine embryos can respond to BMP2 stimulation during initial 3 d of culture in vitro with an increase in blastocyst mRNA for *NANOG* and *CDX2* measured 4 d later and demonstrate effects of BMP2 treatment on indices of cell allocation mediated well after treatment administration. The mechanisms responsible for increased blastocyst *NANOG* and *CDX2* mRNA in response to BMP2 stimulation are not known. However, BMP4 mediated induction of *CDX2* mRNA expression [24] and promoter activity [25] have been described in other cell lines and in human ES cells and signaling for both BMP2 and BMP4 is mediated via SMAD1/5 [16]. However, SMAD2/3 pathways are linked to *NANOG* promoter regulation in embryonic stem cells and antagonistic to BMP signaling and differentiation to TE fate [26]. While the addition of BMP2 had no effect on the proportion of embryos that cleaved early or developed to the 16C or blastocyst stages, the highest dose of BMP2 did increase both *NANOG* and *CDX2* mRNA compared to control embryos at the blastocysts stage. NANOG is a marker of the ICM of the blastocysts and is important for maintaining pluripotency of the cells in the ICM [27]. In contrast CDX2 is a marker for TE cells and is in fact required for the establishment of the TE in the blastocysts of mice and cattle [28,29]. NANOG and CDX2 also cross regulate each other to promote proper blastocyst formation [30]. As BMP2 supplementation increases mRNA for both markers it is possible that BMP2 enhances the differentiation of the two cell types, rather than promoting one cell type over the other.

## Conclusions

In summary, results of present studies demonstrate pronounced temporal regulation of mRNA for select BMP ligands, type I and II receptors and cognate intracellular signaling molecules during bovine early embryonic development. This study was limited in that it only examined changes in abundance rather than accompanying changes in protein abundance. However, to our knowledge, temporal changes in abundance of mRNA for specific BMP including *BMP2*, *BMP3*, *BMP7* and *BMP10* at specific stages of bovine early embryonic development have not been examined previously. While stimulatory effects of BMP2 on early cleavage and development to 8C to 16C and blastocyst stages were not noted in response to BMP2 supplementation of culture media (at doses tested) during first 72 h of development, increased mRNA for *CDX2* and *NANOG* was detected in resulting blastocysts, demonstrating a functional BMP2 signaling system in early bovine embryos. Furthermore, distinct results observed in present studies suggest that embryotrophic actions of follistatin reported in previous studies [3] likely are not linked to inhibition of endogenous BMP2 activity.

## Competing interests

The authors declare that they have no competing interests.

## Authors’ contributions

KBL collected samples, isolated RNA, performed RT-qPCR analysis, performed embryo culture experiments and contributed to design of studies and interpretation of results. JKF analyzed data and contributed to design of studies, interpretation of results and helped draft the manuscript. SR performed RT-qPCR analysis and contributed to design of studies and interpretation of results. GWS conceived the study, contributed to design of experiments and interpretation of results and helped draft the manuscript. All authors read and approved the final manuscript.

## Supplementary Material

Additional file 1: Figure S1 Temporal changes in RPS18 mRNA during oocyte maturation and early embryogenesis in vitro. Quantitative real time RT-PCR analysis was performed on samples of germinal vesicle (GV) and metaphase II (MII) stage oocytes and in vitro derived embryos collected at the pronuclear (PN), 2-cell (2C), 4-cell (4C), 8-cell (8C), 16-cell (16C), morula (MO) and blastocyst (BL) stages (n = 4 pools of 10 oocytes/embryos per pool). Data are shown as mean ± SEM. Values with different superscripts across time points denote significant differences (P < 0.05). Click here for file
