# Supplementary figures and images for: Temporal regulation of mRNAs for select bone morphogenetic proteins (BMP), BMP receptors and their associated SMAD proteins during bovine early embryonic development: effects of exogenous BMP2 on embryo developmental progression
Source: Reprod Biol Endocrinol. 2014 Jul 15;12:67. doi: 10.1186/1477-7827-12-67 (PMC4110370; doi:10.1186/1477-7827-12-67)

# RPS18

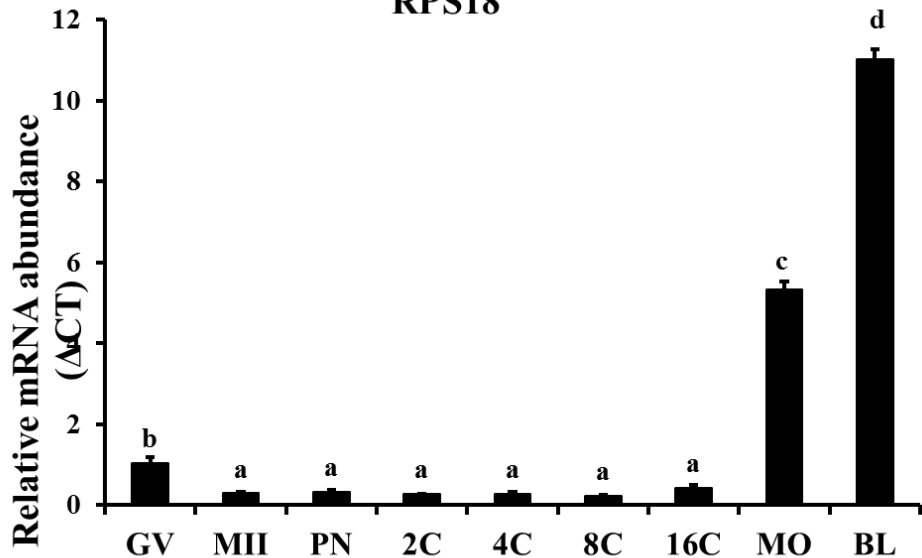

Supplement: Additional file 1: Figure S1 — Temporal changes in RPS18 mRNA during oocyte maturation and early embryogenesis in vitro. Quantitative real time RT-PCR analysis was performed on samples of germinal vesicle (GV) and metaphase II (MII) stage oocytes and in vitro derived embryos collected at the pronuclear (PN), 2-cell (2C), 4-cell (4C), 8-cell (8C), 16-cell (16C), morula (MO) and blastocyst (BL) stages (n = 4 pools of 10 oocytes/embryos per pool). Data are shown as mean ± SEM. Values with different superscripts across time points denote significant differences (P < 0.05). [file 1477-7827-12-67-S1.pdf]
